# Monoamine Oxidase Contributes to Valvular Oxidative Stress: A Prospective Observational Pilot Study in Patients with Severe Mitral Regurgitation

**DOI:** 10.3390/ijms251910307

**Published:** 2024-09-25

**Authors:** Raluca Șoșdean, Maria D. Dănilă, Loredana N. Ionică, Alexandru S. Pescariu, Monica Mircea, Adina Ionac, Cristian Mornoș, Constantin T. Luca, Horea B. Feier, Danina M. Muntean, Adrian Sturza

**Affiliations:** 1 Department VI—Cardiology, “Victor Babeș” University of Medicine and Pharmacy of Timișoara, E. Murgu Sq. no 2, 300041 Timișoara, Romania; sosdean.raluca@umft.ro (R.Ș.); pescariu.alexandru@umft.ro (A.S.P.); adina.ionac@gmail.com (A.I.); mornos.cristian@umft.ro (C.M.); constantin.luca@umft.ro (C.T.L.); 2 Research Centre of the Institute of Cardiovascular Diseases, G. Adam Str. no 13A, 300310 Timișoara, Romania; mircea.monica@yahoo.ro (M.M.); horea.feier@gmail.com (H.B.F.); 3 Department III—Pathophysiology, “Victor Babeș” University of Medicine and Pharmacy of Timișoara, E. Murgu Sq. no 2, 300041 Timișoara, Romania; daninamuntean@umft.ro (D.M.M.); sturza.adrian@umft.ro (A.S.); 4 Centre for Translational Research and Systems Medicine, “Victor Babeș” University of Medicine and Pharmacy of Timișoara, E. Murgu Sq. no 2, 300041 Timișoara, Romania; ionica.loredana@umft.ro; 5 Department X—Medical Semiotics I, “Victor Babeș” University of Medicine and Pharmacy of Timișoara, E. Murgu Sq. no 2, 300041 Timișoara, Romania; 6 Department VI—Cardiovascular Surgery, “Victor Babeș” University of Medicine and Pharmacy of Timișoara, E. Murgu Square no 2, 300041 Timișoara, Romania; 7 “Pius Brinzeu” Emergency County Hospital, 300723 Timisoara, Romania

**Keywords:** severe mitral regurgitation, monoamine oxidase (MAO), reactive oxygen species (ROS), angiotensin 2, angiotensin receptor blocker (ARB), MAO inhibitors (MAOI)

## Abstract

Monoamine oxidases (MAOs), mitochondrial enzymes that constantly produce hydrogen peroxide (H_2_O_2_) as a byproduct of their activity, have been recently acknowledged as contributors to oxidative stress in cardiometabolic pathologies. The present study aimed to assess whether MAOs are mediators of valvular oxidative stress and interact in vitro with angiotensin 2 (ANG2) to mimic the activation of the renin–angiotensin system. To this aim, valvular tissue samples were harvested from 30 patients diagnosed with severe primary mitral regurgitation and indication for surgical repair. Their reactive oxygen species (ROS) levels were assessed by means of a ferrous oxidation xylenol orange (FOX) assay, while MAO expression was assessed by immune fluorescence (protein) and qRT-PCR (mRNA). The experiments were performed using native valvular tissue acutely incubated or not with angiotensin 2 (ANG2), MAO inhibitors (MAOI) and the angiotensin receptor blocker, irbesartan (Irb). Correlations between oxidative stress and echocardiographic parameters were also analyzed. Ex vivo incubation with ANG2 increased MAO-A and -B expression and ROS generation. The level of valvular oxidative stress was negatively correlated with the left ventricular ejection fraction. MAOI and Irb reduced valvular H_2_O_2._ production. In conclusion, both MAO isoforms are expressed in pathological human mitral valves and contribute to local oxidative stress and ventricular functional impairment and can be modulated by the local renin–angiotensin system_._

## 1. Introduction

Valvular heart diseases are highly prevalent and are increasingly recognized as an important public health problem. Mitral regurgitation is the second most common valvular disorder worldwide after aortic stenosis, affecting over two million people [1,2]. Severe mitral regurgitation is a debilitating disease with significant possible complications unless surgically treated on time before permanent damage occurs. If referred late, as sometimes occurs in daily practice for various reasons (patients’ choice and/or slow symptom progression compared to aortic stenosis), patient prognosis is impaired even with the correct treatment [3]. Identifying novel mechanisms that may contribute to disease pathogenesis is worthwhile in order to develop preventive strategies and/or delay its progression.

Oxidative stress is a major pathomechanism of cardiovascular diseases, particularly in aging populations [4], and the main four sources widely acknowledged as being responsible for the increased generation of reactive oxygen species (ROS) are NADPH oxidases, a dysfunctional mitochondrial electron transport system, uncoupled endothelial NO synthase and xanthine oxidase [5,6,7,8,9].

Monoamine oxidases (MAOs) are enzymes bound at the outer mitochondrial membrane in almost all mammalian tissues that catalyze the oxidative deamination of endogenous (e.g., catecholamines) and exogenous (alimentary) amines [10]. Aldehydes, ammonia and hydrogen peroxide (H_2_O_2_) are excessively produced as byproducts of the reaction when MAO catalytic activity is increased and/or the enzyme is overexpressed [11,12]. In humans, MAO-B is the predominant isoform in the brain, while MAO-A is found in the heart and blood vessels [11,13,14]. In practice, there is an armamentarium of available MAO inhibitors (MAOI) to counteract the negative consequences of MAO activation/overexpression, which progressively move from irreversible MAOI (clorgyline for MAO-A and selegiline for MAO-B) to reversible (moclobemide for MAO-A and lazabemide for MAO-B) ones; the latter is devoid of the well-known side-effects of MAO activation [15,16].

In the past two decades, it has become evident that MAOs are important contributors to oxidative stress in the cardiovascular system. Several landmark experimental studies in rodents performed by the groups of Fabio Di Lisa and Angelo Parini unequivocally demonstrated that both MAO isoforms are involved in myocardial ischemia/reperfusion injury and heart failure progression (excellently reviewed in Refs. [12,17,18,19]). In a sophisticated study, the group of Ethan Anderson recently reported that mice with cardiomyocyte-specific MAO-A deficiency showed a significant reduction in incidence and duration of catecholamine stress-induced ventricular tachycardia vs. wild-type mice [20].

Angiotensin 2 (ANG2) is a crucial contributor to the pathophysiology of cardiovascular disease via several mechanisms, one of them being the augmentation of oxidative stress [21,22]. A pioneering study first reported the interaction between MAO and ANG2 in the nervous system, namely, that ANG2 increased MAO activity in rat hypothalamic neurons [23]. More recently, in the cardiovascular system, an ANG2-related increase in MAO expression in mice aorta [11] and human mammary arteries [24] and MAO activity in rat ventricular cardiomyocytes and HL-1 cell lines [25] have been reported.

The literature data regarding MAO roles in the development and progression of valvular heart disease are scarce. As such, MAO-dependent serotonin degradation increased in patients with aortic valve stenosis [26]. We recently reported enzyme expression in an explanted mitral valve in a case of hypertrophic obstructive cardiomyopathy subjected to repair surgery [27] and decided to further analyze it in this pilot study.

In the present study, we tested whether MAOs are expressed and contribute to oxidative stress in mitral valve explants obtained from patients undergoing surgery for severe primary mitral regurgitation and various comorbidities. Further, the ex vivo MAO-ANG2 interaction and its modulation to mitigate oxidative stress, as well as correlations of the latter with clinical and echocardiographic parameters, were also evaluated.

## 2. Results

The present pilot study included 30 patients diagnosed with severe primary mitral regurgitation and hospitalized for surgical intervention (mitral valvular repair and/or replacement). All patients were symptomatic, with hypertension being the most common comorbidity, followed by dyslipidemia, atrial fibrillation, obesity and coronary artery disease, and only sporadic cases with diabetes mellitus and/or chronic kidney disease. Accordingly, patients were further divided into two subgroups: a high-comorbidity group (at least three of the comorbidities mentioned below) and a low-comorbidity group (less than three comorbidities).

The patients’ preoperatory clinical data are summarized in Table 1.

The patients’ preoperatory laboratory results are presented in Table 2. No patient had anemia, infection and/or acute liver disease before surgical intervention.

The patients’ preoperatory echocardiographic parameters are shown in Table 3. The patients had severe primary mitral regurgitation with fibroelastic deficiency/myxomatous degeneration, chordae rupture (*n* = 14, 46.66%) and age-related degeneration (n = 16, 53.33%) etiology in almost equal percentages. Eleven patients (36.66%) had an LVEF less than 55%.

**Figure 1:**
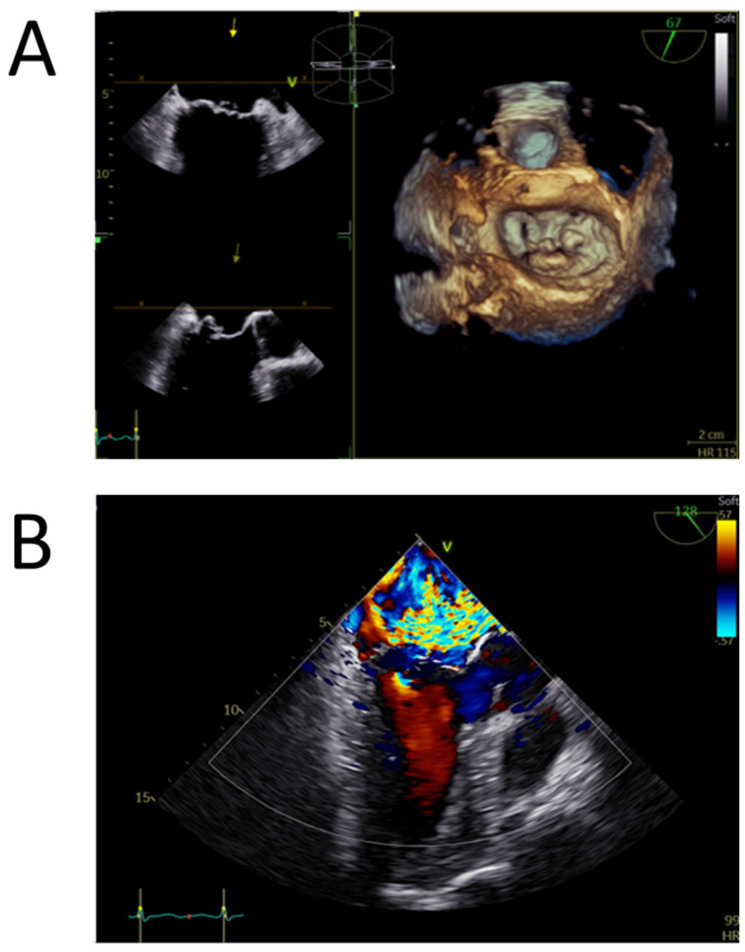
Transoesophageal echocardiography of a myxomatous degenerated mitral valve (Barlow disease). (**A**) Tridimensional model of the mitral valve positioned in the surgical view obtained by zoom 3D acquisitionextensive multisegmental degeneration, with P2 flail and P1 prolapse. (**B**) Mid-oesophageal long axis view with color Doppler revealing a wide eccentric, interatrial septum-directed, mitral regurgitant jet, with a large convergence area compatible with severe mitral regurgitation.

Regarding the mitral valve evaluation, the assessment of the disease severity was best performed using color Doppler during transesophageal echocardiography, whereas the best morphological data were offered by tridimensional transoesophageal echo offline reconstructions (Figure 1). These data were required for the correct diagnosis and decision-making to repair and/or replace the valve.

### 2.1. Both MAO-A and -B Isoforms Are Present and Their Expression Increase in Response to ANG2 in Pathological Human Mitral Valves

**Figure 2:**
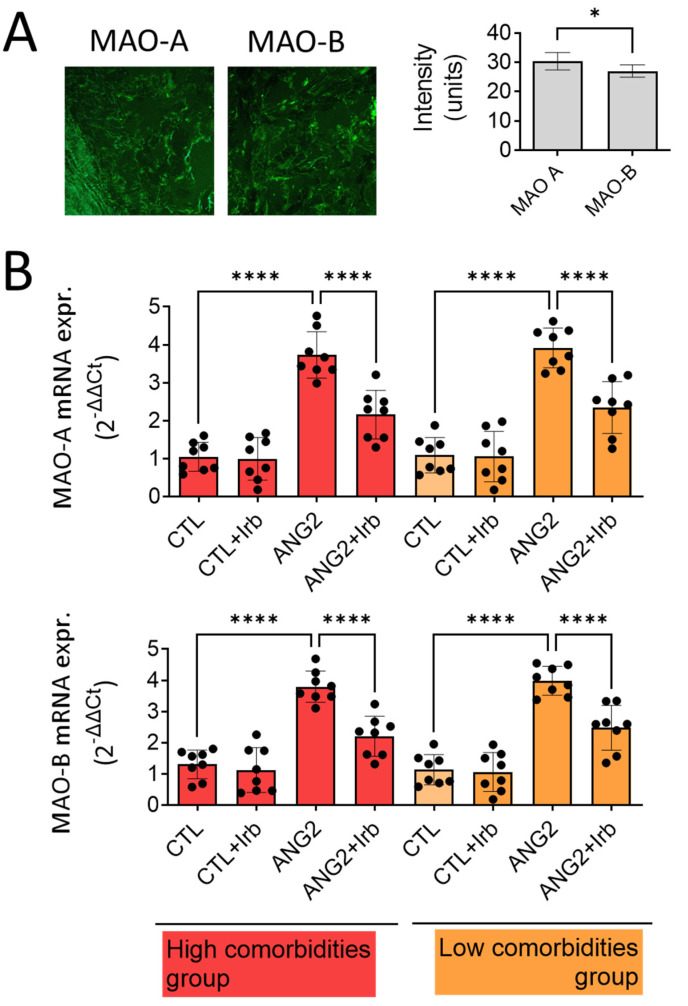
Expression of MAO-A and -B in human mitral valves harvested from high- and low-comorbidity subgroups of patients. (**A**) Immune fluorescence for MAO-A and MAO-B proteins; (**B**) RT-PCR for MAO-A and MAO-B mRNA expression in human mitral valves after stimulation with ANG2 (100 nM, 12 h) in the presence/absence of Irb (10 µM), n = 30, * *p* < 0.05, **** *p* < 0.0001.

We evaluated the protein expression of MAO-A and -B in mitral valve samples using immune fluorescence (IF). The results show that both MAO isoforms were present, being diffusely distributed in the entire mitral valve section, with the predominance of the MAO-A isoform, according to the IF staining intensity (Figure 2A).

As both MAO isoforms were detected in the pathological valves, we wondered whether MAO expression could be differentially upregulated in the setting of increased valvular oxidative stress in patients with high vs. low comorbidity. To address this aspect, the gene expression of MAO-A and MAO-B was determined by RT-PCR. As for protein detected in IF, the gene expression of MAO-A, as judged from the Ct value difference, was more abundant than MAO-B in the diseased mitral valves. Acute ex vivo incubation of the samples in organic cultures with ANG2 (100 nmol/L) increased the expression of both isoforms by approximately three-fold (Figure 2B), suggesting that valvular MAOs are induced in ex vivo conditions that mimic the activation of the renin–angiotensin system. Of note, the presence of comorbidities did not influence the magnitude of MAO-A overexpression. Moreover, co-incubation with irbesartan (10µM), an angiotensin receptor blocker (ARB), almost completely reversed this effect in both subgroups of patients (Figure 2B).

### 2.2. Angiotensin 2 Increased Valvular ROS Generation, an Effect Mitigated by MAO Inhibitors and the Angiotensin Receptor Blocker (ARB), Irbesartan

**Figure 3:**
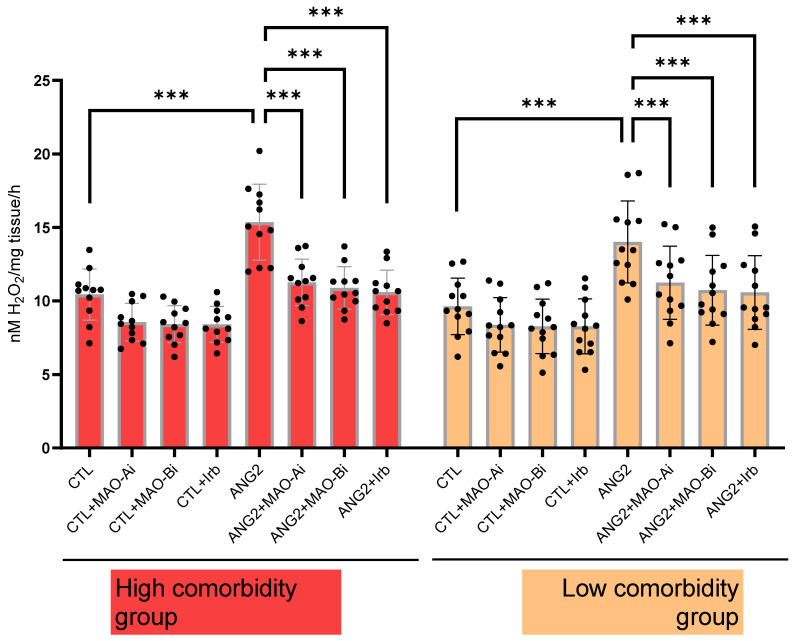
Oxidative stress in explanted mitral valves in high- and low-comorbidity subgroups of patients. FOX assay in mitral valves samples after stimulation with ANG2 (100 nM, 12 h) in the presence vs. absence of an MAO-A inhibitor (clorgyline, 10 µM), MAO-B inhibitor (selegiline, 10 µM) and ARB (irbesartan, 10 µM), n = 30, *** *p* < 0.001.

In order to confirm that ANG2-induced MAO activation contributes to local oxidative stress, H_2_O_2_ generation was measured (with a FOX assay) in valvular samples following acute in vitro incubation with ANG2 (100 nM, 12 h). ANG2 stimulation elicited an increase in the ROS level. The effect was partially reversed upon incubation with either an MAOI (for MAO-A, clorgyline (10 µM) and MAO-B, selegiline (10 µM)) or the ARB, irbesartan (10 µM). The degree of ANG2-induced oxidative stress appeared to be slightly higher in the high-comorbidity subgroup compared to the low-comorbidity one (Figure 3).

**Figure 4:**
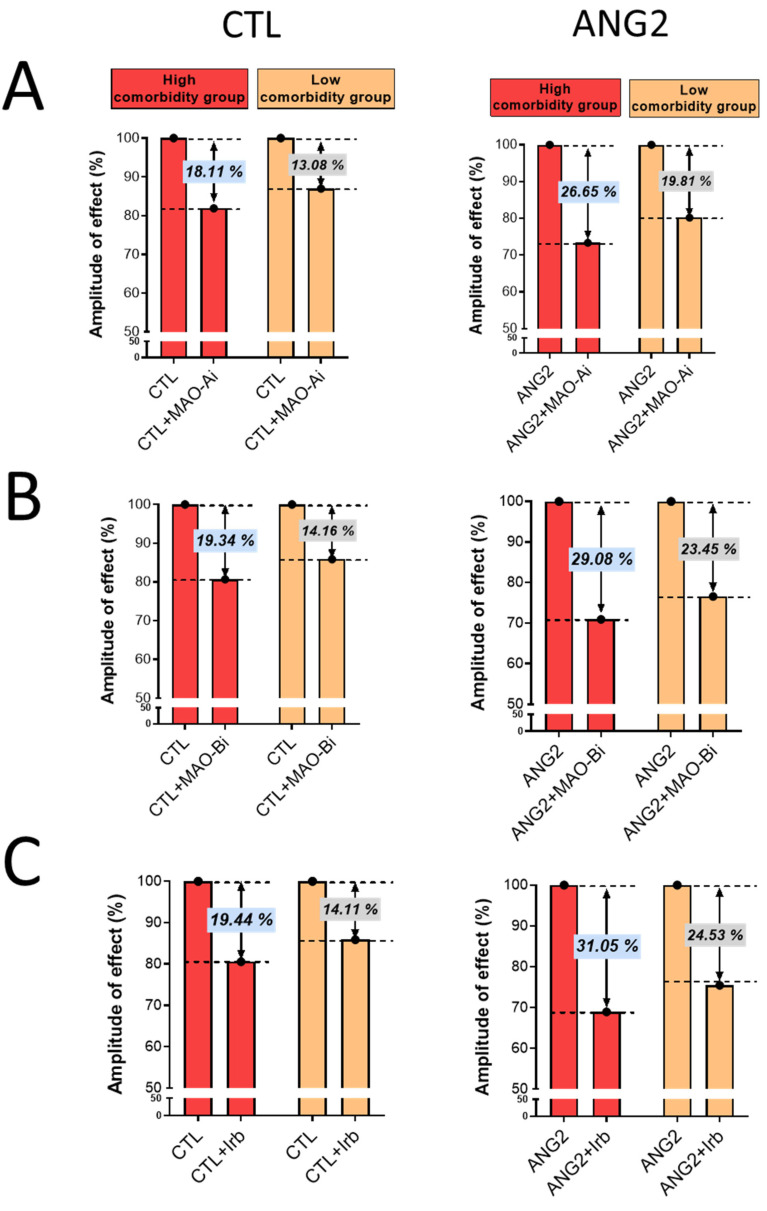
The magnitude of antioxidant effect of MAO-A and -B inhibitors and ARB (Irb) in CTL and ANG2-stimulated valvular samples. (**A**) Effects of MAO-A inhibitors, (**B**) Effects of MAO-B inhibitors and (**C**) Effects of Irb.

Importantly, the magnitude of oxidative stress-lowering effects elicited by incubation with either an MAOI or ARB was more evident in the high-comorbidity subgroup vs. the low-comorbidity one, as depicted in Figure 4.

Collectively, these data demonstrate that MAO-A and -B are contributors to valvular oxidative stress in the presence of ANG2, an effect that is mediated via the type 1 ANG2 receptor since ROS levels decreased after incubation with irbesartan.

### 2.3. Assessment of Correlations between Valvular Oxidative Stress and Clinical and Echocardiographic Parameters

**Figure 5:**
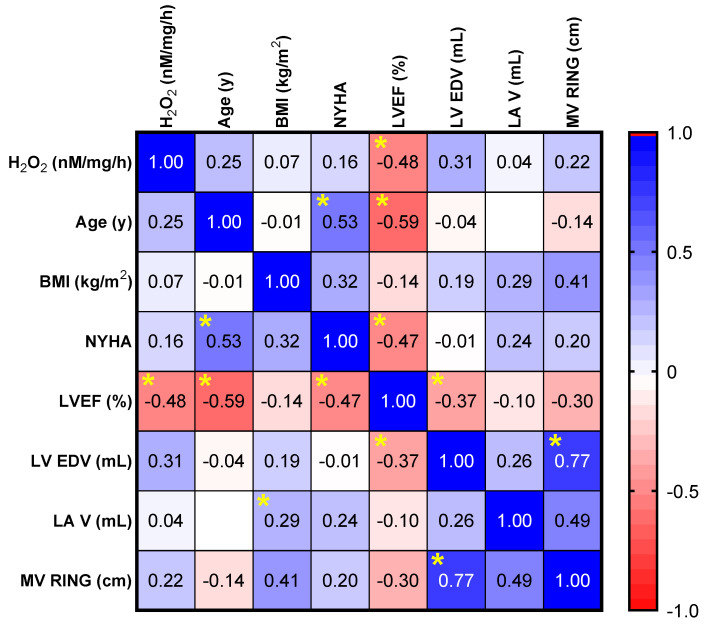
Correlation matrix between the level of oxidative stress (H_2_O_2_ measured by a FOX assay) and the echocardiographic parameters (LVEF, LV EDV, LA V, and MV RING), age, BMI, NYHA degree. * *p* < 0.05.

Correlation analysis was performed to evaluate a potential relationship between the echocardiographic parameters (the LVEF, LV EDV, LA V and MV RING), age, BMI, NYHA class and the degree of oxidative stress (H_2_O_2_ levels were measured by a FOX assay) in the valvular samples. Oxidative stress in mitral valves with severe regurgitation was similar both in age-related degeneration and myxomatous degeneration with chordae rupture (*p* = 0.16). There was also no age-related correlation (R = 0.24, *p* = 0.27) and/or gender-dependent influence (*p* = 0.67). No significant correlation was found between ROS generation and the clinical status of the patient evaluated through the NYHA class for heart failure (R = 0.03, *p* = 0.89). There was no significant difference in valvular oxidative stress in patients with arterial hypertension vs. patients with normal blood pressure (*p* = 0.58). Also, there was no significant correlation between valvular oxidative stress and LV hypertrophy (*p* = 0.14). Similarly, correlations did not have statistical significance either for the left ventricular dimensions (R = 0.35, *p* = 0.1) or for left atrial dimensions (R = 0.04, *p* = 0.85). A significant inverse correlation was found only between oxidative stress (H_2_O_2_ level) and the left ventricular (LV) ejection fraction (R = −0.48, *p* = 0.01) (Figure 5).

## 3. Discussion

The major findings of this pilot study carried out in patients with severe primary mitral regurgitation are as follows: (i) pathological mitral valves express both MAO-A and -B isoforms with a predominance of MAO-A, (ii) MAOs contribute to local oxidative stress, which is potentiated by ANG2, (iii) both MAO expression and oxidative stress are decreased ex vivo by either an MAOI or ARB, an effect that was more prominent in the samples harvested from patients with several comorbidities. Important, all the in vitro experiments were performed without adding external MAO substrates, demonstrating that the damaged valvular tissue contains enough substrates—catecholamines and serotonin—as suggested in the literature [28], which allowed the enzyme activity.

Severe primary mitral regurgitation is a pathology often linked to left atrial enlargement and remodeling, both oxidative stress-related processes. As such, it was reported more than 2 decades ago that atrial stretch may be a trigger for NADPH oxidase activation and subsequent oxidative stress [29]. Since then, the NADPH oxidase (Nox) family has been widely investigated as a major ROS source in the heart, with the NAPDH 4 (Nox4) isoform being the main source of oxidative stress in a failing heart and whose expression has been reported to increase with age. Moreover, complex crosstalk has been reported to occur with other ROS sources in the heart and vessels, thus amplifying oxidative stress and contributing to disease severity/complications [30,31].

Youn et al. highlighted the role of NADPH-related oxidative stress in the pathophysiology of atrial fibrillation [32].

Increased valvular oxidative stress may add to the disease-related increase in ROS production by heart cavities and their remodeling and arrhythmia risk. In a pioneering pilot study (16 patients with severe mitral regurgitation), Chang et al. reported increased activity and expression of a membrane-bound Nox2 containing NADPH oxidase in the atrial appendages, suggesting its contribution to atrial remodeling in these patients [33]. Gladden et al. identified increased xanthine oxidase as a source of oxidative stress as well as clusters of small mitochondria as a hallmark of a bioenergetic defect in the left ventricle of patients with isolated mitral regurgitation and an LVEF > 60% [34]. Hagler et al. demonstrated that the activation of TGF-β signaling is a major contributor to fibrosis and matrix remodeling in myxomatous mitral valve degeneration, amplified by increased oxidative stress [35]. More recently, Songia et al. investigated levels of osteoprotegerin, a molecule linked to oxidative stress and associated with cardiometabolic disorders in patients who underwent mitral valve repair due to mitral valve prolapse. They found that patients with mitral valve prolapse had higher ROS and osteoprotegerin levels than the control group [36].

Studies tackling the role of MAO in the cardiovascular system mainly focused, as mentioned in the introduction, on its role in oxidative stress from ischemia/reperfusion injury, ventricular remodeling/heart failure and vascular dysfunction. Anderson et al. demonstrated that increased MAO activity in right atrial appendages was associated with a high risk of postoperative atrial fibrillation in patients undergoing cardiac surgery [37].

The data in the literature regarding MAO expression/activity in the setting of valvular disease are scarce, and demonstrating its presence and contribution to valvular oxidative stress, although just one source out of several ROS generators, may represent a viable therapeutic target allowing treatment adjustment before clinical aggravation. In our study, MAO expression and ROS production were similar regardless of patients’ cardiovascular and metabolic comorbidities. However, the response to MAO inhibitors and an ARB was more intense in the subgroup with more than three comorbidities and without RAAS inhibition treatment, suggesting that RAAS inhibitors present an antioxidant effect on valvular ROS. Increased ROS production as an effect of ANG2 exposure, as well as the antioxidant effect of an ARB, is concordant with the results of studies tackling other sources of oxidative stress in the setting of heart failure [38]. Our results demonstrate this also holds true for MAO-related oxidative stress in pathological mitral valves.

Nevertheless, MAO induction/activation only partially explains the H_2_O_2_ increase in diseased valves. As such, it has been reported that in rat aortic cells, Nox 4 is responsible for basal H_2_O_2_ production, while superoxide production in both non-stimulated and ANG2-stimulated cells is Nox 1-dependent [39].

A number of studies have addressed the signal transduction of oxidative stress in the setting of valvular diseases. Kruithof et al. analyzed 21 prolapsing mitral valves, demonstrating supplemental valvular thickening through superimposed tissue, in addition to myxomatous degeneration, which was induced mostly on the atrial side by different types of mechanical stress. They showed an association with oxidative stress secondary to the activation of transforming growth factor beta (TGF beta) and bone morphogenetic protein (BMP) in human valves and mouse models [40]. Oyama et al. published a recent comparative study on the pathophysiology of human vs. canine myxomatous mitral valve degeneration, showing that the TGF beta and serotonin pathways are closely related and have a very important role in the evolution of valvular disease. Mechanical stress and serotonin-induced valvular interstitial cell activation with increased transcription and expression of the two aforementioned pathways, resulting in the activation of mitogenic pathways and increased production of the extracellular matrix [41].

Of note, the contribution of serotonin to valvular impairment was also evident in patients with carcinoid tumors of enterochromaffin cells that produce serotonin, which developed valvular pathologies [28]. However, it was the seminal study by Pena-Silva et al. that directly demonstrated the contribution of MAO-A (but not of NADPH oxidase) to ROS production in the homogenates of heart valves from explanted human hearts (not used for transplantation) incubated with serotonin [42].

Among the factors that contribute to increased valvular oxidative stress, a major role is played by the high velocity, high-pressure regurgitant jet that induces mechanical stress on the mitral valves, which, with disease progression, might become more important than the primary valvular disease. Shear stress has been systematically demonstrated to impair redox homeostasis in vascular endothelium, and the four ROS sources are the same as reported in the heart [43] and diseased valves [44].

The mitral valve system is a complex structure that involves mitral leaflets, mitral annulus, chordae tendineae, papillary muscles and the adjacent left ventricle wall. The competency of the mitral valve is dependent on the coordinated interplay of these structures, which makes the etiology of mitral regurgitation varied, with significant consequences for long-term evaluation, management and prognosis [1]. Mitral regurgitation may occur as a result of mitral valve leaflet disease and/or defects in the mitral valve apparatus or as a consequence of left ventricular dysfunction. This condition may be classified as primary or secondary. Primary mitral regurgitation, also known as degenerative or organic regurgitation, is characterized by an impairment of the valvular apparatus, whereas secondary (functional) mitral regurgitation is mainly due to left ventricular remodeling [45]. The underlying pathophysiologic mechanism of degenerative mitral regurgitation in the younger population is most commonly related to valvular tissue alteration resulting in mitral valve prolapse. The spectrum of severity of mitral valve prolapse ranges from fibroelastic deficiency, with thin leaflets and focal prolapse, to Barlow’s disease, with diffusely thickened and redundant leaflets [33]. Mitral annular calcification, a degenerative process that begins in the posterior annulus and extends into the base of the leaflets and subvalvular apparatus, is an increasing cause of mitral regurgitation in the elderly population [34]. Secondary mitral regurgitation occurs when left ventricular dilatation caused by ischemic or nonischemic cardiomyopathy affects leaflet coaptation in a structurally normal valve. On the other hand, a functional component may be associated with the primary pathology in long-lasting severe mitral regurgitation, inducing left ventricular and left atrial dilatation and supplementally increasing mechanical stress on the valve and surrounding structures. Dysfunction and remodeling may result in apical and lateral papillary muscle displacement, which causes leaflet tethering, dilatation and flattening of the mitral annulus, as well as decreased valve-closing forces [46].

In our pilot study, valvular oxidative stress did not show a significant correlation with increasing LA and LV dimensions. This may possibly be due to the low number of patients or the fact that the included patients had similar stages of LA and LV dilatation. They were mostly in the compensated and/or transition stage of mitral regurgitation [47]. The lack of a correlation with the NYHA class may be due to the same reasons. Moreover, patients can often tolerate a progressively decreasing LVEF well compared to acute alterations, which were not included in our study. The significant negative correlation between oxidative stress and a LVEF may be explained by the fact that patients with an already decreased LVEF have a longer history of significant mitral regurgitation and, thus, a longer exposure to significant mechanical stress [48].

Also, hypertension did not elicit a significant influence in our group of patients nor did the associated LV hypertrophy (other causes of concentric LV hypertrophy were excluded in this group), but the results might have been affected by the fact that the majority of the included patients were hypertensive. Also, the antihypertensive medication might have played a role.

It is only a matter of time until a severe mitral regurgitation jet will induce, by volume overload, progressive left atrial dilation with atrial fibrillation as a consequence, progressive alteration of the LV myocardium and a progressive LVEF decrease along with LV dilation. The fact that valvular oxidative stress negatively correlated in our study with an LVEF may be explained by the duration of severe functional alteration. Long-lasting severe mitral regurgitation may be associated with increased values of valvular oxidative stress due to the valvular shear stress induced by the jet for a long period of time. At the same time, the resulting long-lasting volume overload induced by this regurgitation induces LA dilation, eventually with atrial fibrillation and a decreasing LVEF along with LV dilation [49]. It would be interesting to see how early in the valvular morphological and functional evolution oxidative stress is induced and augmented, as once induced, this is a possible mechanism of valvular continuous alteration [40].

In past decades, MAO-mediated H_2_O_2_ production has been systematically reported to contribute to oxidative stress, not only in the heart and vessels, as mentioned in the Introduction, but also in the kidneys [50,51] and adipose tissue, particularly in the presence of inflammation [52] and obesity [53,54].

Maggiorani et al. recently summarized the contribution of MAOs to the pathophysiology of senescence, highlighting the fact that enzymatic expression substantially increases with aging, four-fold for MAO-B in the brain and six-fold for MAO-A in the heart [55]. This observation renders mitochondrial enzymes attractive targets to prevent/treat age-related chronic pathologies based on the fact that new generations of MAO inhibitors are currently used in the clinical arena. Of note, MAO has emerged as a viable therapeutic target since its inhibition does not impair cellular bioenergetics and cellular viability as occurs when inhibiting other well-described ROS generators, particularly the dysfunctional electron transport system at the inner mitochondrial membrane [12].

The present pilot study demonstrates that MAOs, in particular, MAO-A, are sources of oxidative stress in the pathological mitral valvular tissue that contains the substrates required for its activity and that local ROS production can be further increased by ex vivo exposure to ANG2. Whether the increased enzymatic expression is an early event or a later component of a vicious circle of [56], which further exacerbates tissue damage, remains to be established.

### Study Limitations

We acknowledge several limitations of this pilot study, such as the low number of patients that were included in the study after the established indication for valvular surgery (thus, after they were routinely evaluated) and the lack of specific laboratory investigations (e.g., CRP, IL-1 and -6, etc.) that would have been helpful in characterizing the inflammatory status of the patients, as oxidative stress can be aggravated in the presence of low-grade inflammation. We also acknowledge the fact that the technique used to quantify the oxidative stress, the FOX assay, is a simple spectrophotometric method reported in the literature to be suitable for the measurement of low extracellular H_2_O_2_ levels; however, it is not specific, i.e., it will detect other water-soluble hydroperoxides, such as butyl and cumyl hydroperoxides [57]. Also, since the ferrous ion-containing reagent is sensitive to oxidation by atmospheric oxygen, we carefully handled and freshly prepared all the solutions. Last but not least, an accurate analysis of the impact of the treatment regimens that the patients were on before surgery was not possible due to their highly variable doses.

## 4. Materials and Methods

### 4.1. Study Design

This is a prospective single-center randomized cohort study. We included 30 adult patients between 18 and 80 years of age, with severe primary mitral regurgitation and surgical indication (mitral valve replacement and/or repair) according to the current European Society of Cardiology Guidelines for the management of valvular disease [54,55], between March 2020 and July 2021, after signing a detailed and explicit informed consent. Patients with ischemic and/or infectious etiologies, extreme ages, pregnancy, chronic inflammatory diseases, neoplasia, acute kidney injury, chronic end-stage renal disease, a left ventricular ejection fraction (LVEF) below 40% and/or other significant left valvular diseases were excluded, as these situations were considered possible confounders for the study main purpose.

The study complies with the Declaration of Helsinki and was approved by the research ethics committees (the Research Development Ethics Committee of the Institute of Cardiovascular Diseases, Timișoara, Romania, No. 1238/18.02.2020 and the Scientific Research Ethics Committee of “Victor Babeș” University of Medicine and Pharmacy in Timișoara, Romania, No. 02/28.02.2020, respectively). All patients were evaluated in the Cardiology Clinic, and surgical intervention was performed in the Cardiovascular Surgery Clinic, Research Center at the Institute of Cardiovascular Diseases. The valvular tissue sampled during each intervention was introduced in a cold transport media and sent to the laboratory for ex vivo experiments. All the experimental procedures were performed at the Centre for Translational Research and Systems Medicine at “Victor Babeș” University of Medicine and Pharmacy in Timișoara, Romania.

Several cardiometabolic comorbidities that may have an impact on ROS production (chronic kidney disease, diabetes mellitus, arterial hypertension, dyslipidemia, atrial fibrillation, coronary artery disease and clinically advanced heart failure) and echocardiographic parameters (showing a dilated left ventricle and/or reduced ejection fraction), as well as the treatment previously received by the patient, were thoroughly analyzed. After inclusion in the study, patients were divided into two subgroups: a high-comorbidity group (at least 3 from the above-mentioned comorbidities, without pretreatment with RAAS inhibitors) and a low-comorbidity group (less than 3 comorbidities and/or pretreatment with RAAS inhibitors).

### 4.2. Preliminary Patient Evaluation

Clinical and paraclinical evaluation of the patients was performed in a standardized way through detailed anamnesis, physical examination, laboratory tests, transthoracic and transesophageal echocardiography and angiocoronarography as part of diagnostic workup and preoperatory evaluation before inclusion in the study. Data were gathered from patient files and the hospital’s electronic archive.

The echocardiographic evaluation was performed as in daily practice, following the standard protocol and the current European Association of Cardiovascular Imaging recommendations, using General Electric Milwaukee, WI, Vivid 9 and Vivid E 95 echo machines, and offline analysis was performed using the machines’ software and ECHOPAC (version 203) as an external software.

Most of the measurements were performed by transthoracic echocardiography. The left ventricular end-diastolic diameter (LV EDD), interventricular septum (IVS) and left ventricular posterior wall (LVPW) were measured in the two-dimensional parasternal long axis view at the level of the opened mitral valve leaflets’ tips, perpendicular to the LV. Left ventricular concentric hypertrophy was defined by an IVS/LVPW ≥ 12 mm. The left atrium antero-posterior diameter (LA APD) was measured in the same view but in the end-systole at the aortic sinuses’ level, perpendicular to the aortic root [58]

The left ventricular volumes were measured in apical four-chamber and apical two-chamber views using the planimetric biplane technique (tracing the blood–tissue interface), and the left ventricular ejection fraction was subsequently calculated using the Simpson biplane method. Left ventricular dilatation (excentric hypertrophy) was defined by an LV EDV > 75 mL/m^2^. The left atrial volume was calculated in the same manner and incidences [58].

The valvular function was evaluated using Doppler echocardiography (color Doppler as well as a spectral Doppler-pulsed and continuous wave) to verify associated valvular problems.

Severe mitral valve regurgitation was diagnosed by qualitative analysis of the color Doppler regurgitant jet in a parasternal long axis view and apical views (four-, two- and three-chamber views) and visual assessment of the valve’s morphology in the same views. Also, for quantitative measurements, the regurgitant orifice and volume by the PISA method in the apical four-chamber view and vena contracta width measurement in apical four- and two-chamber views were used whenever applicable. Pulmonary vein flow was also used by placing a pulsed-wave Doppler in the superior right pulmonary vein [59].

To better describe the valvular morphology and function, transesophageal echocardiography was also performed, offering important information for establishing the surgical technique. For this purpose, tridimensional echocardiography was also performed. The 3D zoom mode for the mitral valve was the most frequently used [60].

Myxomatous degeneration with at least two affected segments, a dilated ring, segmental prolapse and/or flail were considered Barlow disease, whilst the valves with only one affected segment were considered fibroelastic deficiency [58]. Fibrosed and/or calcified valves were considered to have age-related degeneration, most often associated with other functional alterations, and, thus, were classified as “other etiology”.

Angiocoronarography was performed according to the current ESC guidelines, and coronary artery disease was defined as >50% stenosis on one or more coronary arteries [61].

### 4.3. Tissue Culture

Valvular samples were cleaned and incubated for 12 h at 37 °C in an EBM culture medium (endothelial basal medium with 0.1% bovine serum albumin) in the presence or absence (CTL, control) of angiotensin 2 (ANG2, 100 nM) with or without an MAOI (clorgyline or selegiline, 10 µM) or the angiotensin 2 receptor blocker, irbesartan (Irb, 10 µM). Subsequently, the tissue was embedded in Tissue Tek for immune-histochemistry studies and oxidative stress quantification.

### 4.4. Immune Fluorescence Studies

The tissue expression of MAO was quantified in frozen sections of human mitral valves using MAO-A (Abcam, Cambridge, UK, ab126751) and MAO-B (Abcam, Cambridge, UK, ab175136) primary antibodies and Alexa Fluor labeled secondary goat anti-rabbit antibody (Invitrogen, Waltham, MA, USA, A32731), respectively, as previously described [24]. Nuclear staining was obtained with DAPI (Santa Cruz, Dallas, TX, USA, SC3598). The slides were examined using an Olympus Fluoview FV1000 confocal microscope (Hachioji, Tokyo, Japan). Images were analyzed with ImageJ (version 1.54g), a free open-source image analysis software.

### 4.5. Oxidative Stress Assessment by Means of Ferrous Iron Xylenol Orange Oxidation Assay

Hydrogen peroxide production was assessed in samples of human mitral valves in the presence vs. absence of MAO inhibitors (clorgyline or selegiline, 10 μM, 12 h incubation) and the ANG2 receptor blocker, irbesartan (10 μM, 12 h incubation) using the ferrous iron xylenol orange oxidation (FOX) assay (PeroxiDetect Kit, Sigma Aldrich, Burlington, MA, USA), as previously described [11,14,52]. The principle of the assay is that peroxides oxidize Fe^2+^ to Fe^3+^ ions at acidic pH. The Fe^3+^ ion forms a colored adduct with xylenol orange, which is spectrophotometrically measured at 560 nm. Subsequently, we calculated the production of H_2_O_2_ using a standard curve. The result is expressed in nmol H_2_O_2_/h/mg tissue.

### 4.6. Real-Time Polymerase Chain Reaction (RT-PCR)

All tissues were homogenized using a Tissue Lyser (Qiagen, Hilden, Germany). Total RNA was isolated (Total RNA Mini SI Isolation Spin-Kit, Applichem, Darmstadt, Germany), measured and verified using a Nanodrop 2000 spectrophotometer (Thermo Scientific, Waltham, MA, USA) and used for reverse transcription (Superscript III RT, Invitrogen, Waltham, MA, USA). Primers against MAO isoforms were designed using sequence information from the NCBI database (5′→ 3′): human MAO-A forward: 5′-CTG ATC GAC TTG CTA AGC TAC-3′ and human MAO-A reverse: 5′-ATG CAC TGG ATG TAA AGC TTC-3′. Human MAO-B forward: GAA GAG TGG GAC AAC ATG AC and human MAO-B reverse: CTC CAC ACT GCT TCA CAT AC. The housekeeping gene (EEF2, eukaryotic elongation factor 2) and its primers were as follows (5′→ 3′): EEF2 forward: GAC ATC ACC AAG GGT GTG CAG and EEF2 reverse: GCG GTC AGC ACA CTG GCA TA.

### 4.7. Statistical Analysis

Statistical analysis was performed using Graph Pad Prism 9. The nominal variables were expressed as numbers and percents, whereas the numerical variables were expressed as mean ± standard deviation. The correlation between variables was evaluated using the Pearson correlation coefficient. The comparison between numerical variables was performed using a 2-tailed, type 2 *t*-test. A *p*-value < 0.05 was considered statistically significant.

## 5. Conclusions

Monoamine oxidase isoforms A and B expressed in damaged mitral valves harvested from patients with severe regurgitation contribute to local oxidative stress and may play a role in the pathogenesis and severity of this disease, regardless of etiology and comorbidities. MAO expression, along with ROS production, was upregulated by ANG2 and mitigated by both MAO-A and -B inhibitors and the ARB, irbesartan. The mitigation of the oxidative stress elicited by either an MAOI or ARB was more important in patients with several cardiometabolic comorbidities and the absence of pretreatment with RAAS inhibitors. These results, in particular the latter observation, should be confirmed in larger cohorts of patients with valvular pathology since it might impact their therapeutics and prognosis; early treatment with ARBs (the dose-dependent effects should also be checked) could help in delaying the progression of disease and occurrence of life-threatening complications (advanced heart failure, malignant arrhythmias, etc.) and the timing of surgery. Larger studies addressing MAO-dependent oxidative stress in relation to long-term RAAS inhibition with either an ARB or ACE in other valvular diseases are worth further investigation in order to shed light on novel interactions that can be therapeutically exploited for the benefit of patients.

## Figures and Tables

**Table 1:** Patients’ preoperatory clinical data.

Clinical Parameter	
Age, years, mean ± STD	62.8 ± 12.62
Sex, M/F, n (%)	20 (66.66%)/10 (33.33%)
Body mass index, kg/m^2^, mean ± STD	28.26 ± 4.02
NYHA class	
II	10 (33.33%)
III	20 (66.66%)
Atrial fibrillation, n (%)	11 (36.66%)
Arterial hypertension, n (%)	22 (73.33%)
Diabetes mellitus, n (%)	2 (6.66%)
Chronic kidney disease, n (%)	1 (3.33%)
Coronary artery disease, n (%)	6 (20%)
Obesity, n (%)	10 (33.33%)
Dyslipidemia, n (%)	13 (43.33%)
Smoking, n (%)	5 (16.66%)
Medication	
RAAS inhibitors, n (%)	7 (23.33%)
Beta-blockers, n (%)	20 (66.66%)
MRI, n (%)	21 (70%)
Statins, n (%)	11 (36.66%)
Antidiabetic drugs, n (%)	2 (6.66%)
Insulin, n (%)	0 (0%)

M—male, F—female, RAAS—renin–angiotensin–aldosterone system, MRI—mineralocorticoid receptor blocker, n—number, STD—standard deviation.

**Table 2:** Patients’ preoperatory blood tests.

Blood Test	
Leukocytes, n/mm^3^	7505.33 ± 1841.45
Hemoglobin, g/dL	14.22 ± 1.44
Erythrocytes sedimentation rate, mm/h	13.53 ± 10.33
Creatinine, mg/dL	0.96 ± 0.17
Aspartate aminotransferase, IU/L	23.93 ± 6.9
Alanine aminotransferase, IU/L	26.86 ± 13.03
Fasting blood glucose, mg/dL	101.73 ± 15.88
Total cholesterol, mg/dL	177.8 ± 47.63
LDL cholesterol, mg/dL	110.73 ± 37.11
Triglycerides, mg/dL	155.66 ± 117.71

N—number, IU—international unities. Values are mean ± STD (standard deviation).

**Table 3:** Patients’ preoperatory echocardiographic parameters.

Echocardiographic Parameter	
LV EDD, mm	5.42 ± 0.73
Indexed LV EDD, mm/m^2^	2.83 ± 0.42
LV EDV, mL	156.1 ± 51.55
Indexed LV EDV, mL/m^2^	81.25 ± 26.42
LV EF, %	55.16 ± 8.9
LVPW, mm	1.07 ± 0.16
IVS, mm	1.21 ± 0.26
LA antero-posterior diameter, mm	4.99 ± 0.65
LA volume, mL	131.23 ± 46.16
Indexed LA volume, mL/m^2^	68.01 ± 22.09
Mitral valve ring diameter, mm	4.20 ± 0.61

LV EDD—left ventricular end-diastolic diameter, LV EDV—left ventricular end-diastolic volume, LV EF—left ventricular ejection fraction, LA—left atrium.Values of the echocardiographic parameters are expressed as mean ± STD (standard deviation).

## Data Availability

Data are contained within the article.
